# Mechanism of *Polygala-Acorus* in Treating Autism Spectrum Disorder Based on Network Pharmacology and Molecular Docking

**DOI:** 10.2174/0115734099266308231108112058

**Published:** 2023-11-20

**Authors:** Haozhi Chen, Changlin Zhou, Wen Li, Yaoyao Bian

**Affiliations:** 1 School of Acupuncture-Moxibustion and Tuina, School of Health Preservation and Rehabilitation, Nanjing University of Chinese Medicine, Nanjing, 210023, China;; 2 Jiangsu Provincial Engineering Research Center of TCM External, Medication Development and Application, Nanjing University of Chinese Medicine, Nanjing 210023, China

**Keywords:** Network pharmacology, molecular docking, *Polygala-Acorus*, autism spectrum disorder, drugs and disease, molecular mechanism

## Abstract

**Background:**

Recent epidemic survey data have revealed a globally increasing prevalence of autism spectrum disorders (ASDs). Currently, while Western medicine mostly uses a combination of comprehensive intervention and rehabilitative treatment, patient outcomes remain unsatisfactory. *Polygala-Acorus,* used as a pair drug, positively affects the brain and kidneys, and can improve intelligence, wisdom, and awareness; however, the underlying mechanism of action is unclear.

**Objectives:**

We performed network pharmacology analysis of the mechanism of *Polygala–Acorus* in treating ASD and its potential therapeutic effects to provide a scientific basis for the pharmaceutical’s clinical application.

**Methods:**

The chemical compositions and targets corresponding to *Polygala–Acorus* were obtained using the Traditional Chinese Medicine Systematic Pharmacology Database and Analysis Platform, Chemical Source Website, and PharmMapper database. Disease targets in ASD were screened using the DisGeNET, DrugBank, and GeneCards databases. Gene Ontology functional analysis and metabolic pathway analysis (Kyoto Encyclopedia of Genes and Genomes) were performed using the Metascape database and validated *via* molecular docking using AutoDock Vina and PyMOL software.

**Results:**

Molecular docking analysis showed that the key active components of *Polygala-Acorus* interacted with the following key targets: EGFR, SRC, MAPK1, and ALB. Thus, the key active components of *Polygala-Acorus* (sibiricaxanthone A, sibiricaxanthone B tenuifolin, polygalic acid, cycloartenol, and 8-isopentenyl-kaempferol) have been found to bind to EGFR, SRC, MAPK1, and ALB.

**Conclusion:**

This study has preliminarily revealed the active ingredients and underlying mechanism of *Polygala-Acorus* in the treatment of ASD, and our predictions need to be proven by further experimentation.

## 1 INTRODUCTION

Autism spectrum disorders (ASDs) are a group of neurodevelopmental disorders characterized by socially obstructive communication, a narrow range of interests or activities, and repetitive stereotypical behaviors [1]. Patients diagnosed with ASD often have multiple comorbidities, such as intellectual disability, speech disorder, language disorder, attention deficit hyperactivity disorder, tic disorder, sleep disorder, gastrointestinal dysfunction, anxiety, and epilepsy [2]. Recent epidemic survey data have revealed that the prevalence of ASD is increasing worldwide. According to the screening data released by the US Centers for Disease Control and Prevention in 2021, the prevalence of ASD is as high as 2.3% [3]. In 2020, the results of the first national assessment survey in China showed the prevalence of ASD in children aged 6–12 years to be 0.7%, with a male-to-female ratio of 4.3:1 [4]. ASD has a serious negative impact on children, both physically and mentally, and leads to a high rate of social disability and low possibility of complete spontaneous recovery, which seriously affects their quality of life. Thus, ASD is a public health issue that requires urgent attention.

While there are no records of autism in ancient Chinese medical literature, the core symptoms of ASD include late speech, fetal-period weakness, lack of intelligence, and emotionlessness in the eyes [5]. However, the etiology and pathogenesis of ASD remain unclear. Currently, in Western medicine, a combination of comprehensive interventions and rehabilitative treatments is used for patients with ASD, but outcomes remain unsatisfactory. The Clinical Guidelines for the Treatment of Pediatrics published by the China Association of Chinese Medicine in 2020 recommend oral Chinese medicine as the main treatment for ASD [6]. Traditional Chinese medicine specializes in herbal pairings, and *Polygala* paired with *Acorus* has been widely used in clinical applications [7, 8]. Both of these medicines were first mentioned in the Agriculture God's Canon of Materia Medica. Notably, *Polygala* benefits the intellect and tranquilizes the mind, thus expelling phlegm and opening the orifices. *Acorus* opens the orifices and expels phlegm, waking up the mind and benefiting the intellect. Renowned doctor Mr. JinMo Shi often prescribes *Polygala–Acorus* pairings, which are believed to benefit the kidneys, enhance brain function and intelligence, and promote mental clarity and tranquility, particularly for conditions, such as dementia, memory loss, and indifferent facial expressions [9-14].

**Fig. (1):**
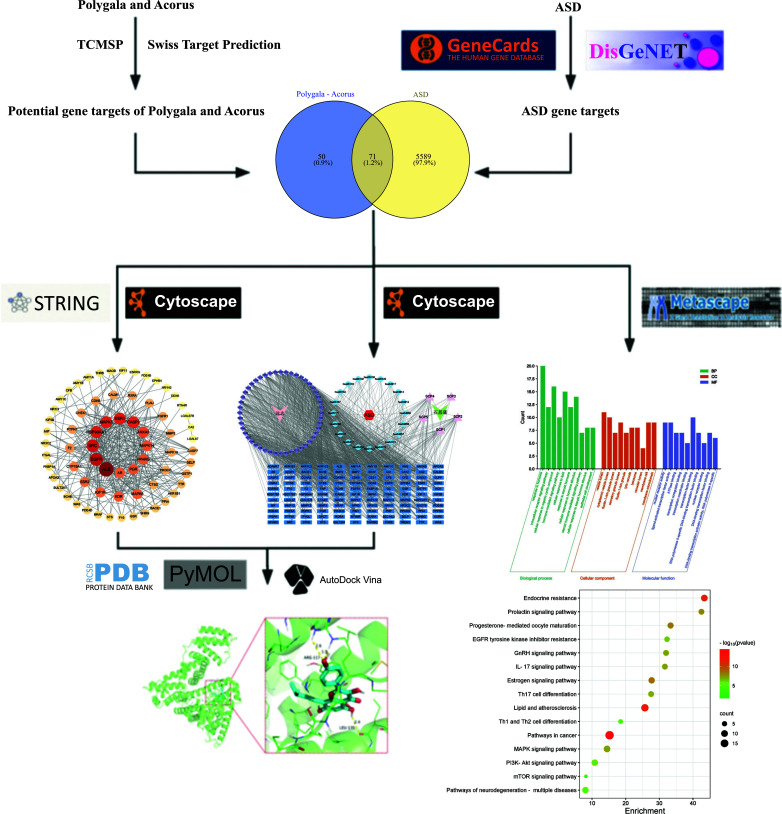
Flowchart of the mechanism of action of *Polygala–Acorus* in the treatment of ASD.

In this study, we performed network pharmacology analysis to evaluate the mechanism of *Polygala-Acorus* in treating ASD (Fig. **1**). This study was conducted to explore and validate the molecular mechanisms and pathways of the main bioactive components of *Polygala-Acorus* in treating ASD. Our results provide a basis for further studies on the pharmacological mechanisms of *Polygala-Acorus* and the potential therapeutic effects of this combination in the clinic.

## 2 MATERIALS AND METHODS

### 2.1 Active Ingredients and Target Points Selection of *Polygala-Acorus*

Using the Traditional Chinese Medicine Systems Pharmacology Database and Analysis Platform (https://old.tcmsp-e.com/tcmsp.php/), the chemical compositions of *Acorus* were searched under the selective condition of oral bioactivity ≥30% and drug-likeness properties ≥0.18 to obtain the chemical substance registration number corresponding to its chemical composition. The Chemical Source Website (https://www.chemsrc.com/) was used to search for *Acorus*, and the CAS number corresponding to its chemical composition was obtained. We used the Chemistry Professional Database of the Shanghai Institute of Organic Chemistry, Chinese Academy of Sciences, to search for *Acorus* and obtain its CAS number. PubChem (https://pubchem.ncbi.nlm.nih.gov/) was used to obtain the CAS numbers of *Polygala–Acorus* corresponding to the 2D structure in SDF format, and the 2D structures were used to obtain compounds corresponding to all individual genes in each herb with the data provided by PharmMapper (http://www.lilab-ecust.cn/pharmmapper/). After aggregation and deduplication, the gene targets of all the active ingredients of *Polygala–Acorus* were obtained for their action in the human body.

### 2.2 Screening of ASD Target Points

We used “autism spectrum disorder” as a search term to obtain aggregative information on the disease targets by searching OMIM (https://omim.org/), DisGeNET (https://www.disgenet.org/), DrugBank (https://go.drugbank.com/), and GeneCards (https://www.genecards.org/). Using the data from these four databases, we obtained the final ASD targets after deduplication.

### 2.3 Access to Drug-disease Intersection Target Points

The drug and disease target points were entered into the online tool Venny (version 2.1.0) (https://bioinfogp.cnb.csic.es/tools/venny/index.html) to obtain the intersection target points.

### 2.4 Protein-protein Interaction Network Construction and Network Topology Analysis

We entered the intersecting target into the String database (https://cn.string-db.org/), chose “multiple proteins” and organism option as “*Homo sapiens*,” under the minimum required interactive sub-selection “medium confidence (0.400)”, and network display selection was performed using “hide disconnected nodes in the network.” Using the default setting for the other options, we obtained protein-protein interaction (PPI) network data. The data were imported into Cytoscape (3.9.1) using the CytoNCA plug-in unit for topological analysis and charting of PPI network.

### 2.5 Gene Ontology Functional Analysis and Kyoto Encyclopedia of Genes and Genomes Metabolic Analysis

To further investigate the specific role of *Polygala–Acorus* in ASD intersecting targets in relevant pathways, we entered the intersecting targets into the Metascape database (https://metascape.org/gp/index.html#/main/step1) with the genetic species option “*H. sapiens*”, and performed Gene Ontology functional enrichment analysis, which included the following three levels: cellular component (CC), molecular function (MF), and biological process (BP); additionally, we performed Kyoto Encyclopedia of Genes and Genomes (KEGG) pathway enrichment analysis.

### 2.6 Selection of Major Active Ingredients and Core Targets

We used Cytoscape to conduct topological analysis while referring to the three main parameters, degree centrality, betweenness centrality, and compact centrality, with the highest parameter considered as the key node. Accordingly, we obtained the gene set of the core targets. Using CytoHubba, we selected maximal clique centrality (MCC) as the optimal algorithm for screening core gene sets. Based on the intersection of the gene set obtained using the two methods above, the main core gene was identified, and its drug component was predicted to be the main active ingredient.

### 2.7 Molecular Docking Verification

We further evaluated the reliability of the interactions between the core components of *Polygala–Acorus* and their corresponding targets. We searched the molecular structures of the main active ingredients obtained from the PubChem database through molecular docking, carried out energy minimization using Chemoffice 3D (2021 version), and saved the results in mol2 format. By searching the UniProt database (https://legacy.uniprot.org/) and setting the limited conditions as validated human-derived genes, we obtained the protein structure of the core genes. Next, we downloaded the protein structure data from the RCSB Protein Data Bank in the RCSB database (https://www.pdbus.org/). To dehydrate and de-ligand the protein molecules using PyMOL, AutoDock tools (1.5.7) were used to dehydrate and hydrogenate the obtained small molecules and protein molecules, and the semi-flexible molecular docking function of AutoDock Vina software was used to set up a grid point with the target protein proto-ligand as the center. Decoding the minimum acquired binding energy between small molecules and target proteins revealed that a smaller numerical value indicated stronger binding. The Lamarckian genetic algorithm was chosen as the docking algorithm, and the rest of the parameters were set by default. To verify the reliability of the AutoDock program for docking in this study system, the original ligand of the target protein complex was abstracted and re-docked to the active pocket of the target protein, and the root-mean-square deviation (RMSD) value of the conformation of the docked ligand from that of the ligand in the original crystal structure was calculated. When the RMSD value is ≤2.0Å, the docking method can better reproduce the original binding pattern of the ligand receptor, which indicates that the docking parameters are set reasonably. The results have been visualized in PyMOL.

## 3 RESULTS

### 3.1 Active Chemical Compositions of *Polygala–Acorus*

Using chemistry data identified by searching the Traditional Chinese Medicine Systems Pharmacology database and Analysis Platform, Chemical Source website, and Chemistry Professional Database Originated from the Shanghai Institute of Organic Chemistry, we obtained 48 active ingredients in *Polygala* and 1120 corresponding targets, with 110 target points remaining after deduplication. *Acorus* contained 5 active chemical components, 103 corresponding target points, and 61 targets remaining after deduplication. The specific active ingredients are listed in Table **1**.

### 3.2 Ways of Operation

By searching for information in the OMIM, DisGeNET, and GeneCards databases using the search term “autism spectrum disorder,” we screened disease targets of ASD; 5660 targets remained after deduplication.

### 3.3 Access to Drug-disease Intersection Targets

We entered the collected drug-disease targets into Venny (version 2.1.0), an online tool, and obtained 71 drug-disease intersection targets. The results are shown in Fig. (**2**), and the specific genes are listed in Table **2**.

### 3.4 PPI Network Construction

**Fig. (4):**
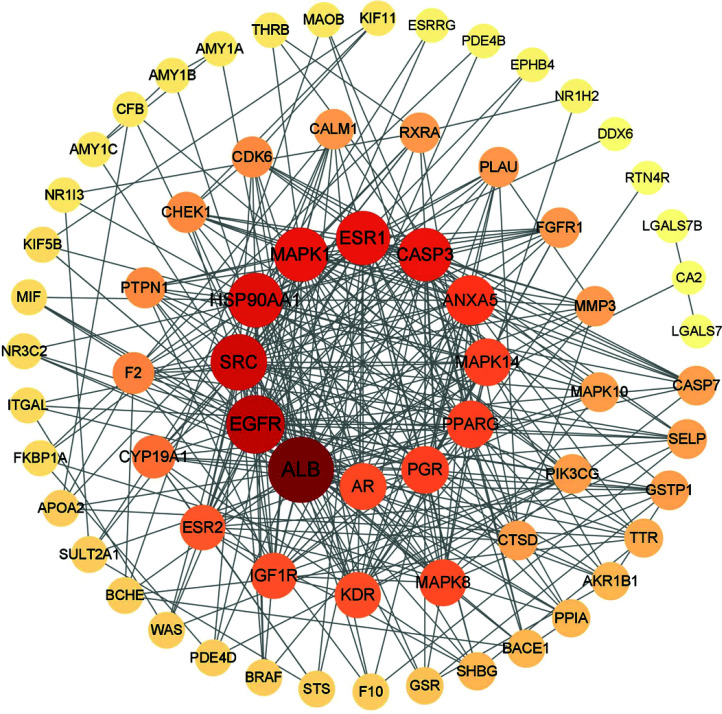
Topological analysis of protein-protein interaction network. A larger circle and darker color indicate a high genetic value.

We inputted the 71 obtained intersection target proteins into the STRING platform, set the confidence degree to medium confidence ≥0.400, and used hidden targets independent of the network to build a PPI network. This network had 71 nodes, 352 edges, and an average node degree of 9.92. The details are shown in Fig. (**3**). We imported the data into Cytoscape (version 3.9.1) and used CytoNCA to topologically analyze the PPI network data. According to the ranked degrees, a larger circle and darker color indicate a high genetic value (Fig. **4**).

### 3.5 GO Function Analysis and KEGG Metabolic Pathway Analysis

The common target points obtained from *Polygala–Acorus* and ASD were entered into the Metascape database, and GO functional and KEGG pathway enrichment analyses were performed. The former analysis revealed 538 BP, 50 CC, and 80 MF processes; the top 10 enriched entries for visual display are shown in Fig. (**5**). Those involving BP enrichment were response to hormones, intracellular receptor signaling pathways, cellular response to organic cyclic compounds, hormone-mediated signaling pathways, cellular responses to lipids, response to steroid hormones, cellular response to hormone stimulus, steroid hormone-mediated signaling pathways, cellular response to steroid hormone stimulus, and epithelial cell development. Further, high-ranked CC enrichment included the vesicle lumen, cytoplasmic vesicle lumen, ficolin-1-rich granule lumen, secretory granule lumen, ficolin-1-rich granules, lytic vacuoles, lysosomes, vacuolar lumen, membrane rafts, and membrane microdomains. Frontal-ranked MF enrichment involved nuclear receptor activity, ligand-activated transcription factor activity, ATPase binding, transcription co-regulator binding, transcription co-activator binding, transcription factor binding, RNA polymerase II-specific DNA-binding transcription factor binding, nuclear receptor binding, DNA-binding transcription factor binding, and DNA-binding transcription activator activity. KEGG analysis showed 140 enriched pathways, and 15 results were chosen for visual display: pathways in cancer, PI3K-Akt signaling, lipid and atherosclerosis, endocrine resistance, progesterone-mediated oocyte maturation, prolactin signaling, MAPK signaling, GnRH signaling, IL-17 signaling, Th17 cell differentiation, pathways of neurodegeneration, multiple diseases, Th1 and Th2 cell differentiation, EGFR tyrosine kinase inhibitor resistance, mTOR signaling, and estrogen signaling. The results are shown in Fig. (**6**).

### 3.6 Screening of Major Active Ingredients and Core Target Points

The CytoNCA results were analyzed using Cytoscape (version 3.9.1) software. The medians of the obtained centrality, betweenness centrality, and compact centrality were 7, 6.354, and 0.247, respectively, and the screened nodes above the medians were considered as key nodes. MCC was used as the optimal algorithm to select the core gene set, the top 10 gene targets were selected by using CytoHubba, and the final core genes (EGFR, SRC, MAPK1, ALB, HSP90AA1, CASP3, ANXA5, ESR1, and MAPK8) were obtained by overlapping the gene sets of the above two methods. The corresponding drug components were considered as the main active ingredients. The details are presented in Table **3**.

The Chinese medicine-active ingredient-core target network contained 197 nodes and 1474 effective relationships. The two V-shaped icons on both sides represent the Chinese herbs *Polygala* and *Acorus*, and the surrounding diamonds and triangles represent the drug ingredients. The red hexagonal icon in the middle represents ASD. Based on the degree of ranking, a darker color indicated the node as more important. 3,6-disinapoylsucrose, bernardioside A, polygalic acid, presenegenin, sibiricaxanthone A, sibiricaxanthone B, tenuifolin, virgaureagenin G in *Polygala* and cycloartenol and 8-isopentenyl-kaempferol in *Acorus* may be crucial components in the treatment of ASD. The corresponding results are shown in Fig. (**7**).

### 3.7 Molecular Docking Verification

**Fig. (8):**
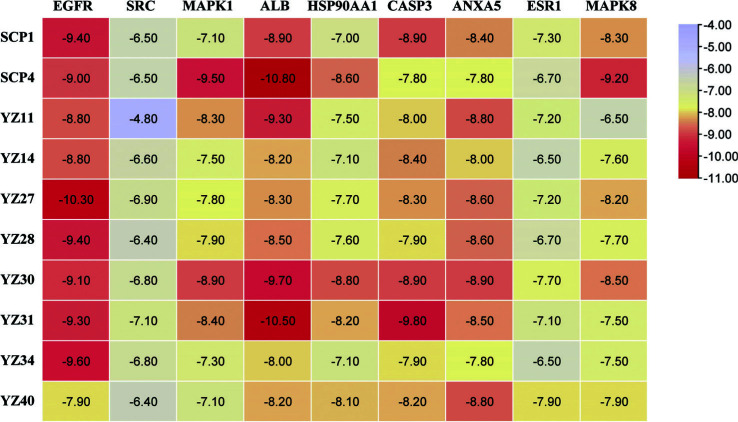
Molecular docking results (kcal·mol^-1^).

Molecular docking of small molecules and target proteins was carried out using AutoDock Vina software. The 10 major compounds of *Polygala-Acorus* were combined with 9 core target proteins, EGFR (PDB ID, 3POZ), SRC (PDB ID, 1O42), MAPK1 (PDB ID, 2OJG), ALB (PDB ID, 1HK4), HSP90AA1 (PDB ID, 1OSF), CASP3 (PDB ID, 1RHJ), ANXA5 (PDB ID, 1HAK), ESR1 (PDB ID, 1SJ0), and MAPK8 (PDB ID, 3ELJ) for docking calculations. The minimum binding energy of small molecules to target proteins showed that a smaller number indicated a stronger binding capacity (Fig. **8**). A docking score binding energy of less than -4.25 kcal/mol was considered to indicate high binding activity between the ligand and target-point proteins. Less than -5.0 kcal/mol indicated better binding and less than -7.0 kcal/mol implied a vibrant docking state between the ligand and target-point proteins [15]. Among the 90 docking results, only one showed a value higher than -5.0 kcal/mol, 13 values were between -6.0 and -7.0 kcal/mol, and the remaining values were below -7.0 kcal/mol. 8-isopentenyl-kaempferol and ALB of *Acorus* showed the highest binding energy of -10.8 kcal/mol; superimposition of the ligand conformation in the original crystal structure of the target protein and the conformation of the ligand after docking clearly showed that the conformation of the ligand after docking overlapped well with that in the original crystal structure, and the RMSD values before and after docking were both 0.013 Å. This value indicated that the present docking method and the docking parameter settings were reasonable and had high credibility. Strong binding was observed between the screened major compounds and core targets. These results provide a basis for further experimental screening and design of herbal medicines. Finally, the first six positions of the docking results were visualized using PyMOL, as shown in Fig. (**9**).

## 4 DISCUSSION

In this study, 48 active ingredients were screened from the traditional Chinese medicine pair *Polygala–Acorus* based on network pharmacology, including 3,6-disinapoylsucrose, bernardioside A, onjisaponin, senegenin, sibiricaxanthone A, sibiricaxanthone B, tenuifolin, polygalic acid, cycloartenol, kaempferol, and 8-prenyl kaempferol. Some studies have shown cycloartenol to be a precursor of phytosterol compounds, which is implicated to have various activities, such as anti-inflammatory, anti-tumor, antioxidant, anti-bacterial, and anti-Alzheimer's disease effects. It also strongly impacts the growth and development of most plants [16]. Kaempferol has been shown to have a neuroprotective effect in rats with cerebral ischemia/reperfusion, possibly *via* its anti-inflammatory, antioxidant, and anti-apoptotic activities [17]. Tenuifolin may exert neuroprotective effects on hippocampal neurons by improving the body's ability to resist oxidative stress, stabilizing mitochondrial membrane potential in the hippocampus, and inhibiting apoptosis [18].

The analysis has revealed *Polygala–Acorus* to mainly involve the nine core genes *EGFR*, *SRC*, *MAPK1*, *ALB*, *HSP90AA1*, *CASP3*, *ANXA5*, *ESR1*, and *MAPK8*. A related study indicated that phosphorylated SRC kinase may boost oligodendrocytes to form myelin sheaths by mediating brain-derived neurotrophic factors, whereas myelin dysplasia can cause multiple complications, such as mental retardation, hearing, and speech impairment [19]. Specific deletion of CASP3 in catecholaminergic neurons leads to hypodopamine function and affects the nigrostriatal dopaminergic pathway, resulting in all core symptoms of ASD [20]. In addition to improving cognitive function, miR-129-5p reduced inflammation in the mouse hippocampus by downregulating MAPK1 expression in chronically mildly stressed mice [21]. miR-132 also improved cognitive function in rats with AD by inhibiting nitric oxide synthase and oxidative stress in the hippocampus by inhibiting MAPK1 expression [22].

The molecular docking results showed that 8-prenyl kaempferol, onjisaponin, and sibiricaxanthone strongly bound to EGFR and ALB. Epidermal growth factor receptor (EGFR) plays a vital role in early brain development, although its expression pattern decreases as the active nervous system matures [23]. However, during neurological decline and brain atrophy, EGFR reappears in brain cells to maintain a balanced neuron bank. It is also involved in regulating learning and memorization, and activated EGFR initiates several signaling pathways simultaneously, such as the PI3K-Akt signaling pathway and MAPK signaling pathway, which are associated with brain plasticity and memory. Disturbance of local receptor tyrosine kinase activity in the brain can affect memory capacity [24].

KEGG analysis showed that the main active components of *Polygala–Acorus* function through the PI3K-Akt signaling pathway, MAPK signaling pathway, interleukin-17 signaling pathway, pathways of neurodegeneration in multiple diseases, EGFR tyrosine kinase inhibitor resistance, mTOR signaling pathway, estrogen signaling pathway, and other pathways. Human recombinant PGRN reduced cerebellar neuronal apoptosis, rescued synapse formation, and protected neurodevelopment in rats with ASD *via* the PI3K/Akt/GSK-3β pathway [25]. Inhibition of mTOR increased PI3K/AKT/mTOR-mediated autophagic activity and improved social interactions in valproate-induced ASD [26]. Another study revealed eIF4E, which is downstream of mTOR, to control and regulate the synthesis of neural proteins and maintain a balance between excitation and inhibition, and that its dysregulation can lead to ASD-like manifestations [27]. Inhibition of the central mTORC1 signaling pathway in the autism model BTBR mice significantly improved social dysfunction and stereotypical behavior, demonstrating that the autistic performance of mice is closely associated with abnormal activation of the mTORC1 signaling pathway [28].

## CONCLUSION

In summary, our study has utilized network pharmacology and molecular docking to predict the key components, target points, and pathways involved in the intervention of ASD by *Polygala–Acorus*. The strong binding activity observed between the core target points and compounds supported the reliability of their network connections. In further studies, we will conduct animal experiments to further evaluate the findings described here.

## Figures and Tables

**Fig. (2):**
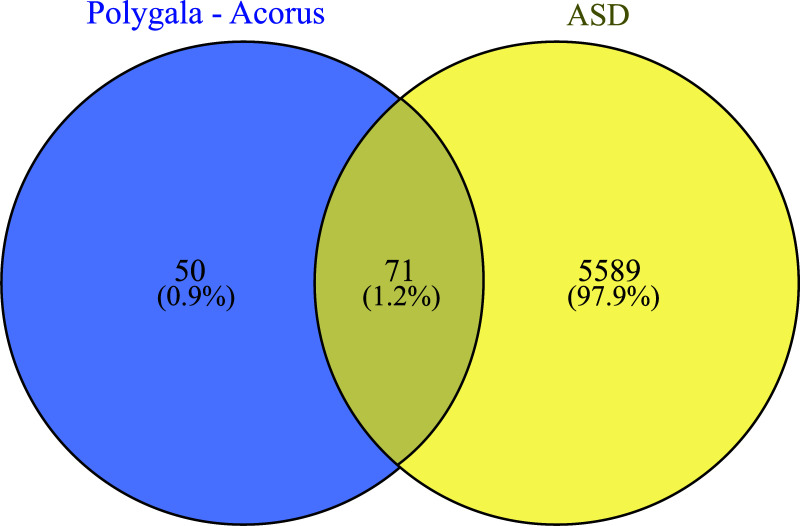
Venn diagram of common genes between *Polygala–Acorus* and autism spectrum disorder.

**Fig. (3):**
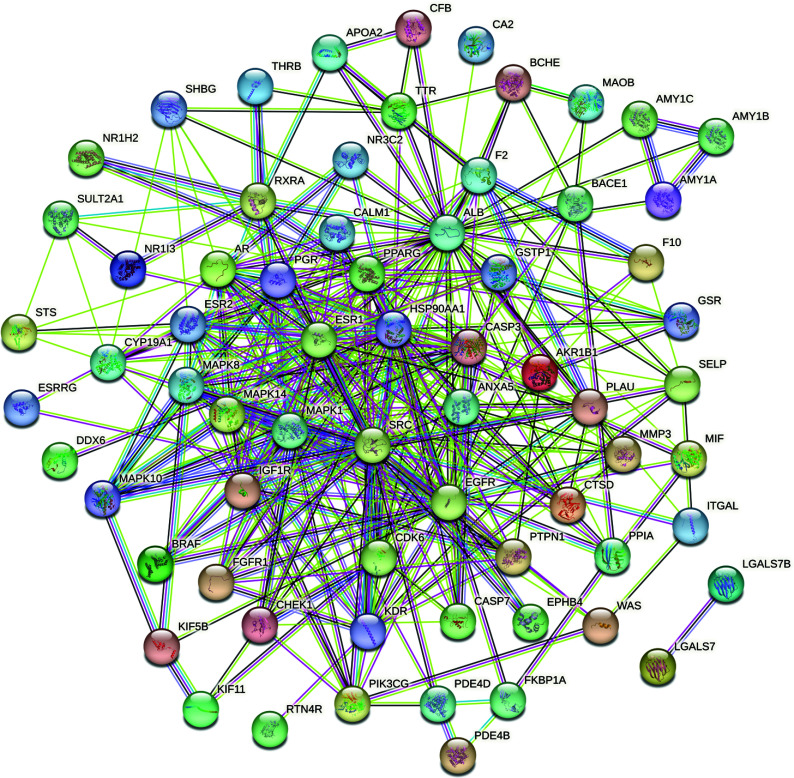
Protein-protein interaction network of potential target genes of *Polygala–Acorus* in treating autism spectrum disorder.

**Fig. (5):**
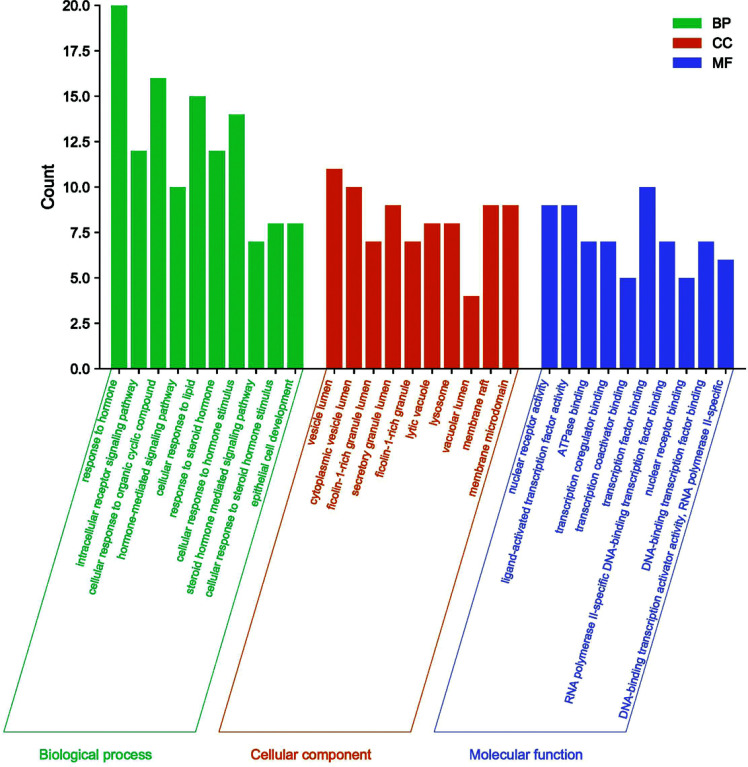
Gene ontology function analysis.

**Fig. (6):**
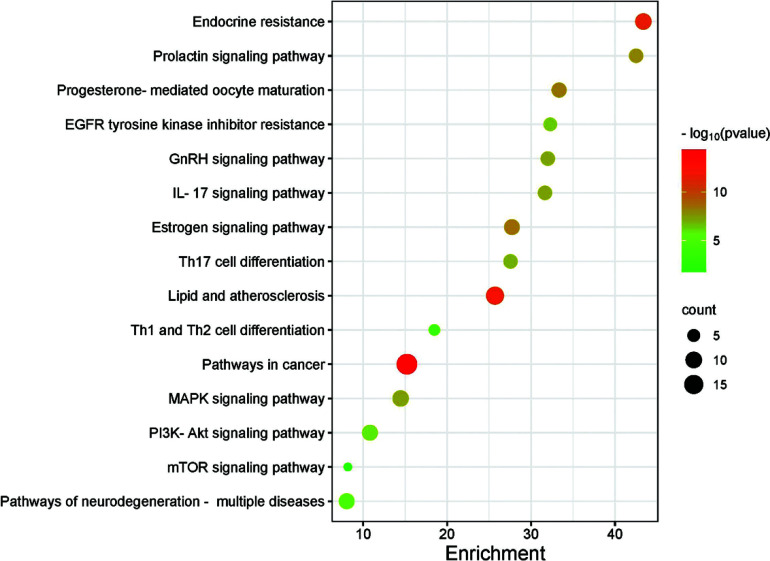
Kyoto encyclopedia of genes and genomes pathway enrichment.

**Fig. (7):**
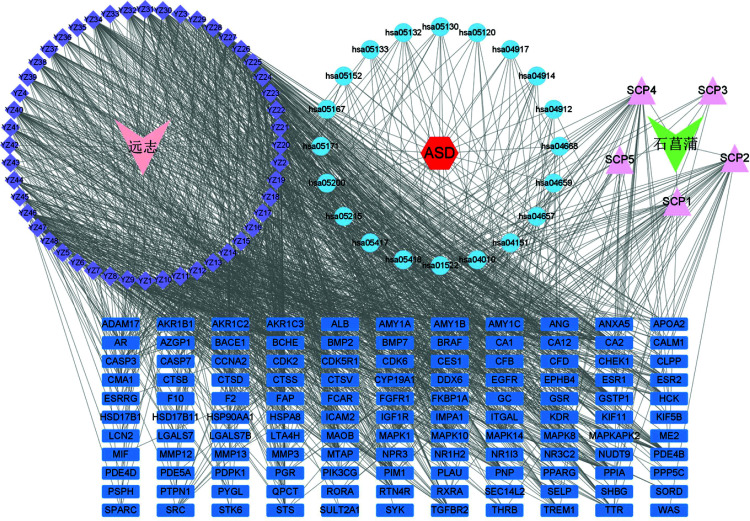
Active components-targets-pathways-disease networks.

**Fig. (9):**
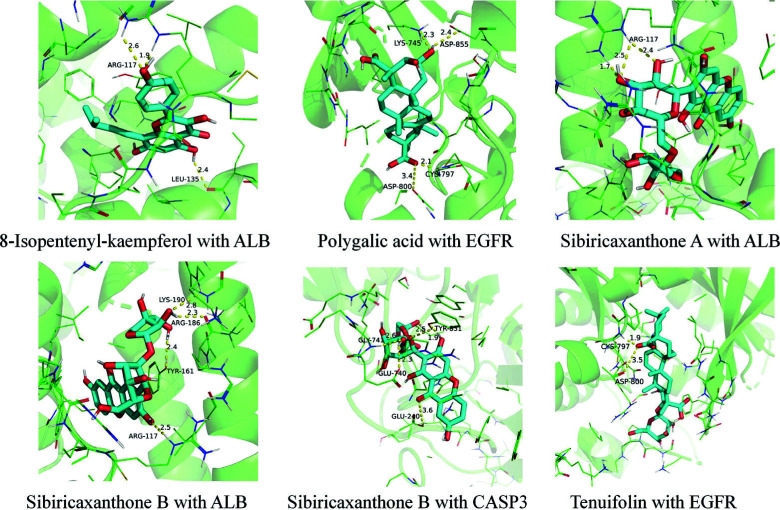
Molecular docking details.

**Table 1:** Active chemical constituents of *Polygala–Acorus.*

**Medicine Name**	**Code**	**Chemical Compound**	**CAS Number**	**PubChem CID**	**Targets**
Polygala	YZ1	1,2,3,6,7-pentamethoxyxanthone	64756-86-1	15693541	3
	YZ2	1,2,3,7-tetramethoxyxanthone	22804-52-0	14528828	11
	YZ3	1,6-dihydroxy-3,5,7-trimethoxyxanthone	65008-17-5	5316837	21
	YZ4	1,7-dihydroxy-2,3-dimethoxyxanthone	78405-33-1	10039726	16
	YZ5	1,7-dihydroxy-3-methoxyxanthone	437-50-3	5281636	8
	YZ6	1,7-dihydroxyxanthone	529-61-3	5281631	9
	YZ7	1,7-dimethoxy-2,3-methylenedioxyxanthone	145523-71-3	85670503	13
	YZ8	1-hydroxy-3,6,7-trimethoxyxanthone	2054-36-6	5318373	11
	YZ9	1-hydroxy-3,7-dimethoxyxanthone	13379-35-6	5488808	11
	YZ10	3,4,5-trimethoxycinnamic acid	90-50-6	735755	7
	YZ11	3,6-disinapoylsucrose	139891-98-8	11968389	37
	YZ12	6-hydroxy-1,2,3,7-tetramethoxyxanthone	64756-87-2	71378875	14
	YZ13	Arillanin A	154287-47-5	11968790	21
	YZ14	Bernardioside A	121368-52-3	14699964	46
	YZ15	Chromeno[4,3-b]chromene-6,7-dione	38210-27-4	441965	15
	YZ16	Ferulic acid	1135-24-6	445858	5
	YZ17	Glomeratose A	202471-84-9	73157754	28
	YZ18	N-acetyl-glucosamine	7512-17-6	1738118	6
	YZ19	Onjisaponin A	82410-33-1	21669941	20
	YZ20	Onjisaponin B	35906-36-6	21669942	27
	YZ21	Onjisaponin F	79103-90-5	10701737	24
	YZ22	Onjisaponin Z	1078708-72-1	134715185	22
	YZ23	Onjixanthone I	136083-92-6	5320290	16
	YZ24	Polygalacin D	66663-91-0	46173909	29
	YZ25	Polygalaxanthone IV	N/A	11972435	43
	YZ26	Polygalaxanthone XI	857859-82-6	101740054	36
	YZ27	Polygalic acid	1260-04-4	12442765	41
	YZ28	Presenegenin	2163-40-8	21594224	43
	YZ29	Senegenin	2469-34-3	12442762	33
	YZ30	Sibiricaxanthone A	241125-76-8	21581292	37
	YZ31	Sibiricaxanthone B	241125-81-5	21581293	36
	YZ32	Sibiricose A6	241125-75-7	6326021	32
	YZ33	Sinapinic acid	530-59-6	637775	5
	YZ34	Tenuifolin	20183-47-5	21588226	51
	YZ35	Tenuifoliose J	147742-14-1	145865805	8
	YZ36	Tenuifoliside A	139726-35-5	46933844	20
	YZ37	Tenuifoliside B	139726-36-6	10055215	34
	YZ38	Tenuifoliside C	139726-37-7	11968391	24
	YZ39	Tenuifoliside D	139726-38-8	5321809	22
	YZ40	Virgaureagenin G	22338-71-2	161388	36
	YZ41	α-spinasterol	54352-47-5	5315190	27
	YZ42	Polygalitol	154-58-5	64960	10
	YZ43	1,6-dihydroxy-3,7-dimethoxyxanthone	69618-09-3	5316766	14
	YZ44	Sibiricose A5	107912-97-0	6326020	26
	YZ45	1,3,6-trihydroxy-2,7-dimethoxy xanthone	136083-93-7	5320291	24
	YZ46	Polygalaxanthone III	162857-78-5	11169063	40
	YZ47	6-O-*p*-hydroxybenzoyl sucrose	139726-39-9	10813903	33
	YZ48	Tenuifoliose A	139682-01-2	145865806	21
Acorus	SCP1	Cycloartenol	469-38-5	92110	31
	SCP2	Kaempferol	520-18-3	5280863	13
	SCP3	Marmesin	13849-08-6	334704	5
	SCP4	8-isopentenyl-kaempferol	28610-31-3	5318624	33
	SCP5	(1*R*,3a*S*,4*R*,6a*S*)-1,4-*bis*(3,4-methoxyphenyl)-1,3,3a,4,6,6a-hexahydrofuro[4,3-c]furan	526-06-7	234823	21

**Table 2:** Specific gene names.

**Gene Names**	**Entry ID**	**Gene Names**	**Entry ID**	**Gene Names**	**Entry ID**
*AR*	P10275	*CHEK1*	O14757	*THRB*	P10828
*BCHE*	P06276	*ESR1*	P03372	*AMY1B*	P0DTE7
*GSTP1*	P09211	*ESR2*	Q92731	*AMY1C*	P0DTE8
*CA2*	P00918	*MAPK1*	P28482	*CASP7*	P55210
*CTSD*	P07339	*PDE4B*	Q07343	*EPHB4*	P54760
*CYP19A1*	P11511	*RTN4R*	Q9BZR6	*SELP*	P16109
*FKBP1A*	P62942	*TTR*	P02766	*ANXA5*	P08758
*PIK3CG*	P48736	*IMPA1*	P29218	*BRAF*	P15056
*PPIA*	P62937	*LGALS7*	P47929	*KIF5B*	P33176
*EGFR*	P00533	*PLAU*	P00749	*PPARG*	P37231
*ESRRG*	P62508	*AKR1B1*	P15121	*PTPN1*	P18031
*GSR*	P00390	*ALB*	P02768	*SULT2A1*	Q06520
*HSP90AA1*	P07900	*APOA2*	P02652	*SRC*	P12931
*PDE4D*	Q08499	*CASP3*	P42574	*NR1I3*	Q14994
*KDR*	P35968	*F10*	P00742	*RORA*	P35398
*MAPK10*	P53779	*F2*	P00734	*MIF*	P14174
*NR1H2*	P55055	*ITGAL*	P20701	*IGF1R*	P08069
*PGR*	P06401	*KIF11*	P52732	*RXRA*	P19793
*BACE1*	P56817	*MAOB*	P27338	*NR3C2*	P08235
*DDX6*	P26196	*MAPK14*	Q16539	*CALM1*	P0DP23
*FAP*	Q12884	*MAPK8*	P45983	*FGFR1*	P11362
*AMY1A*	P0DUB6	*MMP3*	P08254	*CDK6*	Q00534
*CFB*	P00751	*SHBG*	P04278	*WAS*	P42768
*LGALS7B*	P47929	*STS*	P08842		

**Table 3:** Core target points’ properties.

**Targets**	**Name**	**Degree**	**Central Number in Mediation**	**Level of Tight Centrality**
ALB	Albumin	45	1218.3943	0.3018018
EGFR	Epidermal growth factor receptor	35	443.92847	0.28879312
SRC	Proto-oncogene tyrosine-protein kinase Src	32	347.50858	0.2863248
HSP90AA1	Heat shock protein HSP 90-alpha	30	493.08432	0.2838983
ESR1	Estrogen receptor	29	159.86342	0.28033474
MAPK1	Mitogen-activated protein kinase 1	29	291.0724	0.28270042
CASP3	Caspase-3	28	202.23653	0.28033474
ANXA5	Annexin A5	23	70.59635	0.27459016
MAPK8	Mitogen-activated protein kinase 8	19	64.82308	0.26907632

## Data Availability

The data and supportive information are available within the article.

## LIST OF ABBREVIATIONS

ASD: Autism Spectrum Disorder
BP: Biological Process
CC: Cellular Component
EGFR: Epidermal Growth Factor Receptor
GO: Gene Ontology
KEGG: Kyoto Encyclopedia of Genes and Genomes
MCC: Maximal Clique Centrality
MF: Molecular Function
PDB: RCSB Protein Data Bank
PPI: Protein-protein Interaction
